# Systematic review of incidence, risk factors, prevention and treatment of post-laryngectomy hypoparathyroidism

**DOI:** 10.1007/s00405-020-06213-2

**Published:** 2020-07-22

**Authors:** Ovie Edafe, Luke M. Sandler, Nigel Beasley, Sabapathy P. Balasubramanian

**Affiliations:** 1 Department of ENT, Sheffield Teaching Hospitals Foundation Trust, Sheffield, UK; 2 Emergency Department, Croydon University Hospital, London, UK; 3 Oncology and Metabolism, University of Sheffield, Sheffield, UK; 4 Department of General Surgery, Sheffield Teaching Hospitals Foundation Trust, Sheffield, UK

**Keywords:** Laryngectomy, Pharyngectomy, Oesophagectomy, Hypocalcaemia, Hypoparathyroidism

## Abstract

**Purpose:**

Post-laryngectomy hypoparathyroidism is associated with significant short- and long-term morbidities. This systematic review aimed to determine incidence, risk factors, prevention and treatment of post-laryngectomy hypoparathyroidism.

**Methods:**

Medline, EMBASE and the Cochrane library were searched for relevant articles on hypocalcaemia and/or hypoparathyroidism after laryngectomy or pharyngectomy. Two authors independently screened titles and abstracts from the search. Data from individual studies were collated and presented (without meta-analysis). Quality assessment of included studies was undertaken. The review protocol was registered in the PROSPERO database (CRD42019133879).

**Results:**

Twenty-three observational studies were included. The rates of transient and long-term hypoparathyroidism following laryngectomy with concomitant hemi- or total thyroidectomy ranged from 5.6 to 57.1% (*n* = 13 studies) and 0 to 12.8% (*n* = 5 studies), respectively. Higher transient (62.1–100%) and long-term (12.5–91.6%) rates were reported in patients who had concomitant oesophagectomy and total thyroidectomy (*n* = 4 studies). Other risk factors included bilateral selective lateral neck dissection, salvage laryngectomy and total pharyngectomy. There is a lack of data on prevention and management.

**Conclusion:**

Hypoparathyroidism occurs in a significant number of patients after laryngectomy. Patients who underwent laryngectomy with concomitant hemithyroidectomy may still develop hypoparathyroidism. Research on prevention and treatment is lacking and needs to be encouraged.

## Introduction

Post-surgical hypocalcaemia or hypoparathyroidism (PoSH) is a well-documented complication of central compartment neck surgery, particularly thyroidectomy [1]. This is due to direct damage, devascularisation or inadvertent excision of adjacent parathyroid glands.

The reported rates (i.q.r.) of transient and long-term hypocalcaemia following bilateral thyroid surgery were 27% (19–38) and 1% (0–3) [1]. The estimated prevalence of PoSH in Europe and United States of America was 22 per 100,000 and 23 per 100,000, respectively [2]. Risk factors for PoSH following thyroid surgery include inadvertent parathyroid gland excision, identification of fewer than two parathyroid glands, heavier thyroid gland, reoperation for bleeding, surgery for recurrent goitre, retrosternal goitre, Graves’ disease and central neck dissection [1].

PoSH has significant short- and long-term morbidity. Acute hypocalcaemia may present with paraesthesia, neuromuscular irritability, cardiac arrhythmias, and seizures [3]. Clinical features of long-term hypocalcaemia and/or hypoparathyroidism include renal impairment, seizures, neuropsychiatric diseases and infections [4, 5].

A number of preventative measures have been evaluated in thyroid surgery, including the use of routine postoperative supplementation, autotransplantation and haemostatic techniques.

Patients undergoing laryngectomy usually have concomitant unilateral or bilateral thyroidectomy and, therefore, are at risk of developing hypocalcaemia [6]. This is mostly secondary to hypoparathyroidism (risk increased by extent of primary surgery) as other mechanisms of hypocalcaemia such as hungry bone syndrome seen in thyroid surgery do not play a significant role in this scenario. The incidence of transient and long-term PoSH following laryngectomy is not well documented. In addition, risk factors, preventative measures and treatment strategies are not well described.

The aim of this systematic review was to evaluate reported incidence, risk factors, preventative measures and treatment strategies of post-laryngectomy hypocalcaemia or hypoparathyroidism.

## Methods

Medline, EMBASE and Cochrane library databases were searched from the inception of the databases to January 2020 using the key words: (laryngectomy OR pharyngectomy) AND (hypocalc^*^ OR hypoparathyroidism OR low calcium) AND (incidence OR prediction OR risk factors OR preventative measure OR prevention OR treatment OR management OR protocol). All human observational and interventional studies reporting on incidence, risk factors, predictors or management of PoSH following laryngectomy were eligible for inclusion. We excluded case reports, reviews, letters, commentaries, animal studies, and articles not available in English language. The bibliography of included studies was searched to identify additional articles for inclusion.

The main outcomes were the proportion of patients who developed transient and long-term PoSH. The rates of transient and long-term PoSH were reported as defined by individual studies.

Two researchers independently screened titles and abstracts from initial search. Relevant full texts were retrieved and further evaluated for eligibility against the criteria listed above. Data were extracted from included studies using a standardised data collection tool by one reviewer and checked for accuracy by another reviewer. Extracted data included details of study characteristics, population studied, definitions of outcomes and data on any risk factors, preventative methods and management that may be described in the study.

The methodological quality of included studies was assessed using a tool described by Murad et al. [7]. This tool gives a total score of six: based on selection, ascertainment of exposure and outcome, confounding factors, follow-up, and clarity of the report. The quality of articles was assessed as low, medium and high for scores of 0–2, 3–4, and 5–6, respectively.

Quantitative synthesis was not done due to heterogeneity in the extent of surgery and definitions of PoSH used in included studies. Odds ratio (OR) was provided only if reported in the individual studies.

This review was registered with PROSPERO database (CRD42019133879).

## Results

Twenty-three observational studies published between 1968 and 2019 on 1416 patients were included in this review [6, 8–29]. The studies were from Europe (*n* = 11), North America (*n* = 4), Africa (*n* = 4), Asia (*n* = 3), and South America (*n* = 1). Of these, 23 (100%) reported on rates of PoSH, 7 (30.4%) on risk factors, one (4.3%) on prevention and none (0%) on the effectiveness of treatment strategies. There were no interventional studies.

**Fig. 1:**
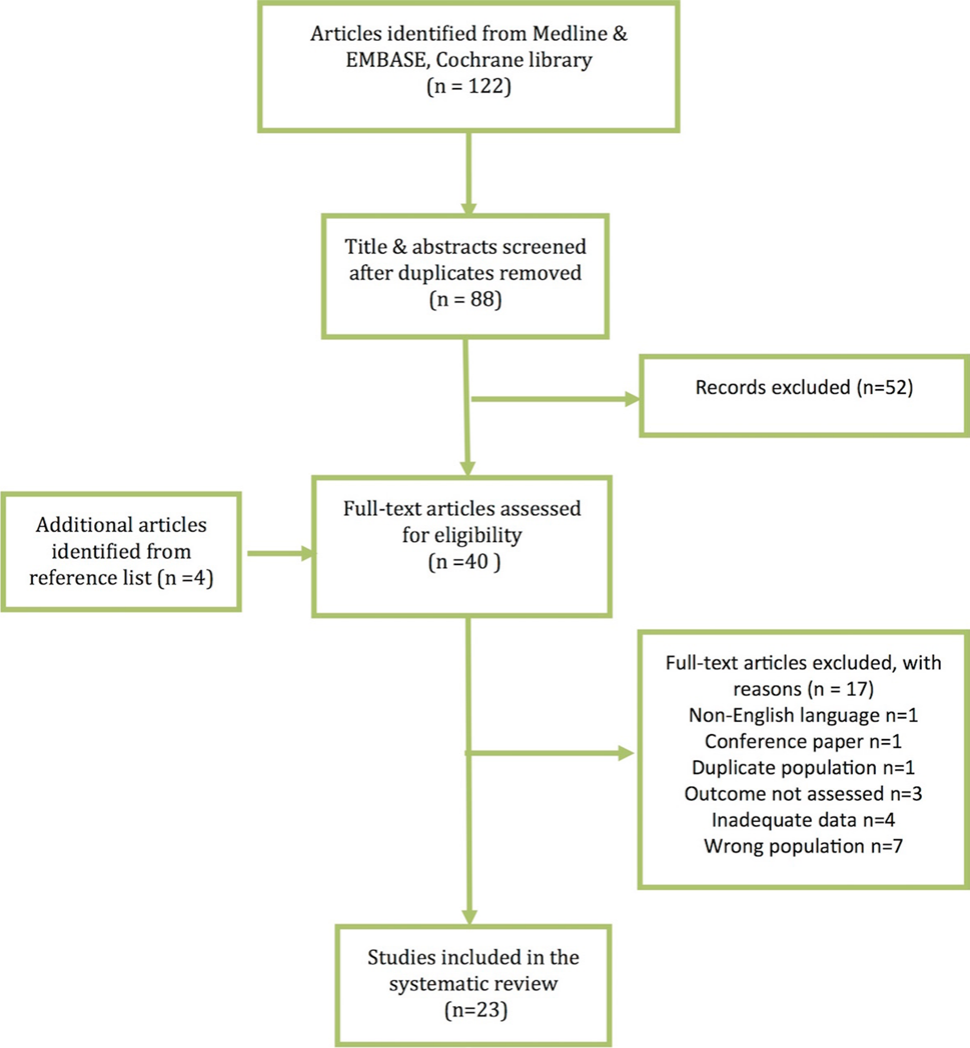
PRISMA flow diagram showing steps of inclusion and exclusion of articles

Figure 1 shows the process of study selection. “Wrong population” represented articles that did not evaluate PoSH following laryngectomy, and “inadequate data” represented articles where the rates of PoSH could not be calculated based on the information provided. The median (i.q.r.) number of patients in the 23 studies was 47 (25–81). Fourteen studies (60.9%) were focused on PoSH, while 9/23 (39.1%) reported on PoSH as a secondary outcome. The median (i.q.r) quality assessment score for the included study was 3 (3.0–3.5). Table 1 shows the quality assessment for each study.

**Table 1:** Quality assessment of included studies

Studies	Q1	Q2	Q3	Q4	Q5	Q6	Total
Farlow et al. [29]	0	1	1	0	1	0	3
Saito et al. [28]	0	1	0	0	1	0	2
Panda et al. [27]	0	1	1	0	1	0	3
Harris et al. [6]	1	1	1	0	1	1	5
Negm et al. [17]	0	1	1	0	1	0	3
Okano et al. [18]	0	1	1	0	1	0	3
Shenson et al. [20]	1	1	0	0	1	0	3
Marion et al. [15]	0	1	0	0	1	0	2
Abd Elmaksoud et al. [8]	0	1	1	1	1	1	5
Basheeth et al. [9]	1	1	1	0	1	1	5
Gurbuz et al. [11]	0	1	0	0	1	0	2
Oosthuizen et al. [19]	1	1	0	0	1	0	3
Leong et al. [13]	1	1	0	0	1	0	3
Lo Galbo et al. [14]	0	1	1	0	1	0	3
Martins et al. [25]	1	1	0	0	1	0	3
Clark et al. [10]	1	1	1	0	1	0	4
Thorp et al. [23]	0	1	1	0	1	0	3
Mortimore et al. [16]	0	1	1	0	1	0	3
Smith et al. [21]	0	1	0	0	1	0	2
Krespi et al. [22]	0	1	0	0	1	0	2
Harrison et al. [12]	1	1	1	0	0	0	3
Buchanan et al. [26]	1	1	1	0	1	1	5
Osborn et al. [24]	1	1	1	0	1	1	5
Median total							3

*Q1* selection methods, *Q2* ascertainment of exposure, *Q3* assessment of outcome, *Q4* alternative causes evaluated, *Q5* adequate follow-up, *Q6* report described in sufficient details

Table 2 shows the characteristics of included studies, definition for PoSH and proportion of patients who had transient and/or long-term PoSH in individual studies. Table 3 gives the reported range of hypocalcaemia stratified by extent of surgery. In patients having total laryngectomy with concomitant hemi/total thyroidectomy, the reported rates of transient and long-term PoSH ranged from 5.6 to 59.3% and 0 to 12.8%, respectively.

**Table 2:** Characteristics of included studies

Studies	Population	Extent of thyroidectomy	Definition of transient hypocalcaemia	Definition of long-term hypocalcaemia	Overall rates of transient (T) and long term (L)
Farlow et al. [29]	Salvage TL	Not reported	Need for calcium supplements and ionised Ca < 1.12 mmol/L	Not reported	T: 20/192 (10.4)
USA					L: 10/144 (6.9%)
* n* = 210					
Saito et al. [28]	Total LPO	Hemi: 17	Not applicable	Need for Calcium/vitamin D or cCa < 8 mg/dL at 3 months after surgery	L: 12/17 (70.6%)
Japan					
*n* = 17					
Panda et al. [27]	TL	Hemi: 104	Not applicable	cCa and need for calcium and vitamin D3 at 12 months	L: 16/125 (12.8%)
India		Near total: 5			
*n* = 125		Total: 16			
Harris et al. [6]	TL	None: 14	cCa ≤ 2.149 mmol/L	Need for calcium/vitamin D > 12 months	T: 16/90 (17.7%)
UK		Hemi 69			L: 10%
*n* = 90		Total: 7			
Shenson et al. [20]	TL	Not reported	Not reported	Not applicable	T: 10/116 (8.6%)
USA					
*n* = 116					
Marion et al. [15]	LP + gastric pull up	Total: 24	Not reported	Not applicable	T: 1/24 (4.2%)
France					
*n* = 24					
Negm et al. [17]	TL	Hemi: 17	cCa < 2.10 mmol/L and PTH < 10 pg/mL	Not applicable	T: 7/17 (41.2%)
Egypt					
*n* = 17					
Okano et al. [18]	Total LPO	None: 2	Intact PTH < 10 pg/ml and serum Ca < 8.5 mg/dL	Not applicable	T: 8/50 (16%)
Japan		Hemi: 38			
*n* = 50		Total: 10			
Abd Elmaksoud et al. [8]	TL: 7x	Total: 26	Serum calcium < 8.0 mg/dL or symptoms	Need for calcium/vitamin D > 6 months	T: 24/24 (100%)
	Total LPO: 19				L: 3/24 (12.5%)
Egypt					
*n* = 26					
Basheeth et al. [9]	TL: 47	Hemi/Isthmus: 39	cCa < 2.00 mmol/L	Not applicable	T: 26/60 (43.3%)
Ireland	TL + total/subtotal P: 13	Total: 21			
*n* = 60					
Gurbuz et al. [11]	TL: 45	Hemi (44)	Not applicable	Not reported	L: 0%
Turkey					
	Supracricoid laryngectomy: 1	Total (3)			
*n* = 47	Near TL:1				
Leong et al. [13]	TL: 61	Not reported	Not reported	Not applicable	T: 4/71 (5.6%)
UK	Total LP: 10				
*n* = 71					
Oosthuizen et al. [19]	TL	Not reported	Not reported	Not applicable	T: 4/30 (13.3%)
Ireland					
*n* = 30					
Lo Galbo et al. [14]	TL	Hemi: 132	Serum calcium < 2.20 mmol/L	> 6 months after surgery	T: 43/137 (31.4%)
The Netherlands					
*n* = 137					L 7/137 (5.1%)
Clark et al. [10]	LP: 145	Not reported	cCa < 2.00 mmol/L requiring treatment	Not applicable	T: 45%
Canada	P: 8				
*n* = 153					
Martins et al. [25]	Total LPO	None: 3	Clinical signs and symptoms of hypocalcaemia	Not applicable	T: 27/40 (67.5%)
Brazil		Partial: 25			
*n* = 40		Total: 12			L: 22/40 (55%)
Thorp et al. [23]	TL ± partial P	Hemi: 20	cCa < 2.10 mmol/L	Not applicable	T: 7/20 (35%)
South Africa					
*n* = 20					
Mortimore et al. [16]	TL: 19	Hemi: 30	Not reported	Not applicable	T: 2/30 (6.7%)
	TL + partial P: 11				
South Africa					
*n* = 30					
Smith et al. [21]	TL: 20	Hemi: 19	Not reported	Not applicable	T: 16/27 (59.3%)
UK	Total LP: 7	Total: 8			
*n* = 27					
Krespi et al. [22]	TL: 17	Not reported	Need for postoperative supplementation	Not applicable	T: 24/47 (51.1%)
USA	Total LPO: 30				
*n* = 47					
Harrison et al. [12]	Total LPO	Total: 58	cCa < 2 mmol/L	Not applicable	T: 36/58 (62.1%)
UK					
*n* = 58					
Buchanan et al. [26]	Total LPO	Total: 6	cCa < 2.00 mmol/L	Not applicable	T: 5 patients
UK					
*n* = 6					
Osborn et al. [24]	TL: 13	Isthmus: 9	Serum calcium < 8.6 mg/dL	Not applicable	T: 7/15 (46.7%)
UK	TL + partial P: 2	Hemi/isthmus: 6			
*n* = 15					

*TL* total laryngectomy, *P* pharyngectomy, *LP* laryngopharyngectomy, *LPO* laryngopharyngo-oesophagectomy, *cCa* corrected calcium, *PTH* parathyroid hormone

**Table 3:** Rates of transient and long-term hypocalcaemia by surgical extent

Extent of surgery^a^	Transient (%)	Long term (%)
Laryngectomy with hemithyroidectomy or total thyroidectomy	5.6–59.3 [6, 9, 10, 13, 14, 16, 17, 19–21, 23, 24, 29]	0–12.8 [6, 11, 14, 27, 29]
Laryngectomy and hemithyroidectomy	6.7–47.3 [6, 9, 14, 16, 17, 21, 23]	0–7 [6, 14, 27]
Laryngectomy and total thyroidectomy	43–87.5 [6, 9, 21]	60–68.8 [6, 27]
Laryngectomy with oesophagectomy and hemithyroidectomy	2.6–70.6 [18, 25, 28]	44 [25]
Laryngectomy with oesophagectomy and total thyroidectomy	62.1–100 [8, 12, 18, 25]	12.5–91.6 [8, 25]

^a^Note some patients had partial/total pharyngectomy

### Extent of primary surgery

Harris et al. compared the rates of transient and long-term PoSH in laryngectomy patients who had total thyroidectomy versus hemithyroidectomy or thyroid preservation [6]. Total thyroidectomy significantly increased the rates of transient (OR 15.5, 95% CI 2.2, 181.9) and long term (OR 22.7, 95% CI 1.9, 376.5) PoSH. Interestingly, Basheeth et al. found no statistically significant difference in the rates of transient PoSH between concomitant total thyroidectomy (57%) versus lobectomy/isthmusectomy (35.9%) in 60 patients following laryngectomy [9].

Clark et al. explored the relationship between pharyngectomy and transient PoSH in patients undergoing laryngopharyngectomy and flap reconstruction. In their multivariable analyses, circumferential pharyngectomy significantly increased the rates of transient PoSH compared to partial pharyngectomy (*P* = 0.017; OR 3.91). In addition, laparotomy to do a free jejunum flap or gastric transposition (compared to fasciocutaneous flaps) was associated with increased risk of transient PoSH [10]. Basheeth et al. found no association between total/subtotal pharyngectomy and transient PoSH in a population undergoing laryngectomy for laryngeal or hypopharyngeal cancer [9].

In the context of laryngo-pharyngo-oesophagectomy for cervical oesophageal cancer, Saito et al. found total oesophagectomy (compared to partial) increased the risk of long-term PoSH [28].

Furthermore, previous tracheostomy prior to laryngectomy was not found to be associated with transient PoSH [9].

### Extent of nodal surgery

Basheeth et al. also evaluated the association between selective lateral neck dissection and transient POSH in 60 patients undergoing laryngectomy. In their multivariable analysis, they found bilateral selective neck dissection increased the risk of transient PoSH (OR 4.56, 95% CI 1.26, 15.80) [9].

Two studies evaluated paratracheal lymph node dissection and transient PoSH [14, 29]. Lo Galbo et al. evaluated complication associated with paratracheal neck dissection in 137 patients who had laryngectomy with or without hemithyroidectomy [14]. They excluded patients who had total thyroidectomy as part of the primary operation. They found bilateral paratracheal lymph node dissection did not significantly increase the rates of transient PoSH compared to unilateral paratracheal lymph node dissection (50% versus 50%). Farlow et al. examined the effects of paratracheal neck dissection in 210 patients who underwent salvage laryngectomy [29]. They found no significant difference in rates of transient PoSH in patients who had bilateral PTLN, unilateral PTLN, and no PTLN (17% versus 6% versus 8%, respectively). In addition, the rates of long-term PoSH were not significantly different between the groups.

### Primary versus salvage surgery

Three studies evaluated the association between salvage laryngectomy and transient PoSH [9, 10, 28]. Basheeth et al. found salvage laryngectomy increased the risk of transient PoSH in their multivariable analysis (OR 4.83, 95% CI 1.17–20.00). Clark et al. examined morbidity after flap reconstruction in tumour involving the hypopharynx. In their univariable analysis, they found no statistical significant difference in the rates of hypocalcaemia between primary (51%) and salvage (40%) surgery [10]. Another study of 17 patients with cervical oesophageal tumour found no association between chemoradiotherapy treatment and hypoparathyroidism following surgery [28].

### Tumour stage

One study evaluated the association between tumour stage and hypoparathyroidism in 60 patients undergoing laryngectomy [9]. They found pT4 stage tumour stage was not associated with transient PoSH.

### Postoperative radiotherapy

In a study of 17 patients, Negm et al. found no association between postoperative radiotherapy and transient PoSH. They excluded patients who had total thyroidectomy as part of laryngectomy [17].

### Prevention

The effect of parathyroid gland autotransplantation on hypocalcaemia was studied in 24 patients who underwent laryngectomy with total thyroidectomy [8]. The parathyroid glands were excised, stored in ice, and following histological confirmation and exclusion of malignancy were autotransplanted onto the anterior forearm muscles. The patients received routine calcitriol (25–50 ug) and calcium carbonate (2–3 g). All developed transient hypocalcaemia within 5 days postoperatively. At 6 months, three patients (12.5%) were on calcium and vitamin D supplements.

### Treatment

There was no study evaluating the effectiveness of treatment strategies or management protocol on PoSH following laryngectomy.

## Discussion

To our knowledge, this is the first systematic review on hypocalcaemia or hypoparathyroidism following laryngectomy.

This review aimed to ascertain reported rates of hypocalcaemia or hypoparathyroidism following laryngectomy and to identify risk factors, preventive measures and treatment strategies described in the literature. Contrary to thyroid surgery, patients who underwent laryngectomy with hemithyroidectomy may still develop transient and long-term hypocalcaemia regardless of the preservation of the contralateral thyroid lobe. However, the reported rates of transient and long-term hypocalcaemia were higher in patients who had total thyroidectomy (Table 3). Transient hypocalcaemia can occur in up to 100% of patients who had total laryngectomy with concomitant oesophagectomy and total thyroidectomy [8, 18, 25]; long-term hypocalcaemia is also higher in this group [25].

The definition of transient and long-term PoSH varied between individual studies as seen in Table 2. Some definitions only required reduction in serum calcium levels [6, 9, 12, 14, 23, 24, 26], while others required reduction in both calcium and PTH levels in defining hypocalcaemia [17, 18]. Corrected serum calcium levels were generally used but one study used ionised calcium levels [29]. The duration for the definition of long-term PoSH was specified as 6 months in some studies [8, 14] and 12 months in others [6, 27]. Some studies reported rates of transient and long-term hypocalcaemia without providing a definition in the manuscript [11, 13, 15, 16, 19–21].

The British Association of Thyroid and Endocrine surgeons (BAETS) defined transient post-thyroidectomy hypocalcaemia as adjusted serum calcium < 2.10 mmol/L on day 1 postoperatively [30]. Although this cut-off could be used to standardise the definition of hypoparathyroidism post-laryngectomy, patients have longer inpatient stay following laryngectomy and limiting the definition to day 1 would underestimate the rates of transient hypoparathyroidism. An alternative standard definition could be the lower limit of postoperative PTH levels which have been shown to be a reliable indicator of transient PoSH [31] and predictor of long-term PoSH [32].

To define long-term hypocalcaemia and hypoparathyroidism, the need for calcium/and or vitamin D supplement to maintain normocalcaemia is recommended by the BAETS [30]. The European Society of Endocrinology Clinical Guideline also used 6-month duration to diagnose long-term hypoparathyroidism; however, their definition includes a low PTH level [33]. Standardising definitions would enable a fair comparison of PoSH rates across units and in the literature. In thyroid surgery, the rates of postoperative hypocalcaemia have been shown to vary between 0 and 46% in a cohort depending on the definition used; highlighting the important of standardised definitions [34].

The risk factors for transient hypocalcaemia identified in this review (total thyroidectomy [6], bilateral neck dissection [9], salvage total laryngectomy [9], total pharyngectomy [10], free jejunum flap [10], and gastric transposition [10]) reflect the extent of surgery involved. Risk factors for PoSH in thyroid surgery such as inadvertent parathyroid gland excision and intraoperative identification of fewer parathyroid glands have not been studied in patients undergoing laryngectomy.

Only one study reporting on preventative measures demonstrated that routine autotransplantation of parathyroid glands may prevent long-term PoSH in patients who had en bloc resection of thyroid and parathyroid glands with total laryngectomy [8]. Parathyroid gland autotransplantation is well reported in thyroid surgery. Other preventative measures evaluated in thyroid surgery include routine peri-operative calcium and vitamin D supplementation, intraoperative parathyroid identification and use of haemostatic devices (including harmonic scalpel, ligasure) [35]. As in thyroid surgery, preoperative identification and treatment of vitamin D deficiency may help reduce the incidence and severity of post laryngectomy hypocalcaemia. Furthermore, the use of fluorescent imaging [36] and electric impedance spectroscopy [37] has been studied to aid intraoperative parathyroid localisation. These novel preventative measures may guide preservation and direct autotransplantation.

There were no studies on management strategies or treatment protocols for PoSH following laryngectomy. Management of hypocalcaemia following laryngectomy is challenging in the early postoperative phase, particularly in patients with gastric pull up reconstruction. These patients appear to require higher doses of calcium supplementation to normalise serum calcium [22]. In addition, careful monitoring of serum calcium is required to prevent iatrogenic hypercalcaemia [38]. As reported in the literature on thyroid surgery [39, 40], implementation of standard protocols on the identification and early treatment of PoSH after laryngectomy may reduce short-term morbidity, reduce hospital stay and the need for intravenous calcium supplementation.

The systematic review is limited by the sparsity of available data in low to moderate quality case series. There was variability in extent of surgical intervention and the definitions used for transient and long-term PoSH; which precluded meta-analysis.

Further well-designed cohort studies are required to further characterise hypocalcaemia following laryngectomy as good quality data on his complication are lacking. The association between inadvertent parathyroid excision and hypocalcaemia in this group has not been explored. Further work is also needed to identify and evaluate preventative measures. In addition, studies on treatment protocols to guide management are also required.

## Conclusion

Hypocalcaemia following laryngectomy has been reported in a significant proportion of patients. There are limited data on preventative measures and treatment strategies for post-laryngectomy hypoparathyroidism.
